# Symptomatic eating epilepsy: two novel pediatric patients and review of literature

**DOI:** 10.1186/s13052-021-01051-2

**Published:** 2021-06-12

**Authors:** Fabiana Vercellino, Laura Siri, Giacomo Brisca, Marcello Scala, Antonella Riva, Mariasavina Severino, Pasquale Striano

**Affiliations:** 1 Child Neuropsychiatry Unit, SS. Antonio e Biagio e Cesare Arrigo Hospital, Spalto Marengo 46, 15121 Alessandria, Italy; 2 Child Neuropsychiatry Unit, “IRCCS Istituto Giannina Gaslini”, Genoa, Italy; 3 Subintensive Care Unit, “IRCCS Istituto Giannina Gaslini”, Genoa, Italy; 4 Pediatric Neurology and Muscular Disease Unit, IRCCS Istituto Giannina Gaslini”, Genoa, Italy; 5 Department of Neurosciences, Rehabilitation, Ophthalmology, Genetics, Maternal and Child Health (DINOGMI), University of Genoa, Genoa, Italy; 6 Neuroradiology Unit, “IRCCS Istituto Giannina Gaslini”, Genoa, Italy

**Keywords:** Eating epilepsy, Reflex seizures, Symptomatic epilepsy

## Abstract

Eating epilepsy (EE) is a form of reflex epilepsy in which seizures are triggered by eating. It is a rare condition but a high prevalence has been reported in Sri Lanka. In EE, the ictal semiology includes focal seizures with or without secondary generalization or generalized seizures. Some cases are idiopathic while focal structural changes on imaging, if present, are often confined to the temporal lobe or perisylvian region. On the other hand, some cases support the hypothesis of a genetic aetiology. The prognosis of EE is extremely variable due to the different nature of the underlying disorder. We describe two patients with symptomatic eating epilepsy, a 13-year-old boy with a bilateral perisylvian polymicrogyria and a 2-year-old boy with a genetic cause. The presence of structural lesions or the dysfunction of specific cortical regions in the context of a germline genetic alteration might lead to a hyperexcitation fostering the epileptogenesis. We review the available literature to clarify the aetiopathogenesis and the mechanisms underlying EE to improve the diagnosis and the management of these rare conditions.

## Background

Eating epilepsy (EE) is characterized by recurrent reflex seizures provoked by eating. Although a high prevalence of eating epilepsy has been previously reported in Sri Lanka, it is considered to be a rare condition in the rest of the world [1]. The exact mechanism underlying this type of seizures remains unknown. Diverse mechanisms related to eating such as gastric distension, mastication and swallowing, and chemical composition of food have been proposed as possible stimuli [2, 3]. While most cases are idiopathic, focal structural changes involving the temporal lobe or perisylvian region have been described [4]. Besides, the observation of familial eating epilepsy supports the hypothesis of a genetic aetiology [1, 5]. In EE, the ictal semiology mostly consists of focal seizures with or without secondary generalization [6]. Monotherapy or combination therapy is generally necessary. However, therapeutic options for medically refractory seizures are not well-established. We describe two patients with symptomatic eating epilepsy, and review eating epilepsy in the available literature.

## Case presentation

The patients were observed between 2014 and 2018. The EE diagnosis was clinical and based on patient history and electroencephalogram (EEG), genetic analysis and magnetic resonance imaging (MRI) data.

### Patient 1

**Fig. 1:**
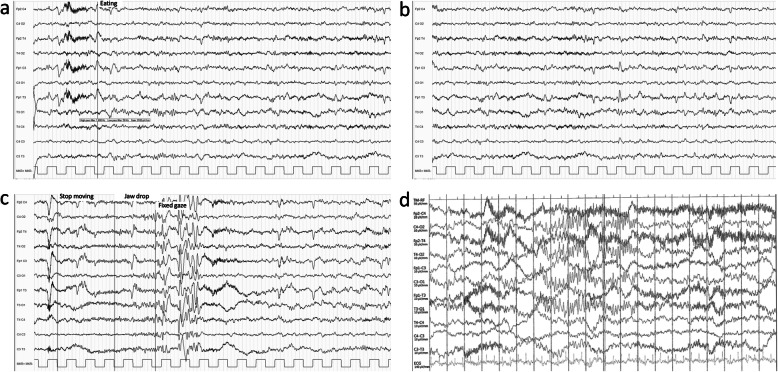
Ictal EEG recordings of patient 1 (13 years) after 10″ (**a**) and 30″ (**b**) from food intake; showing a brief seizure characterized by jaw hypotonia and gaze staring associated with diffuse low-amplitude fast activity followed by generalized spikes and sharp waves mainly evident on mid-temporal areas (**c**). Ictal EEG of patient 2 showing a brief discharge of high-voltage spikes and sharp waves mainly evident on mid-temporal areas (**d**)

**Fig. 2:**
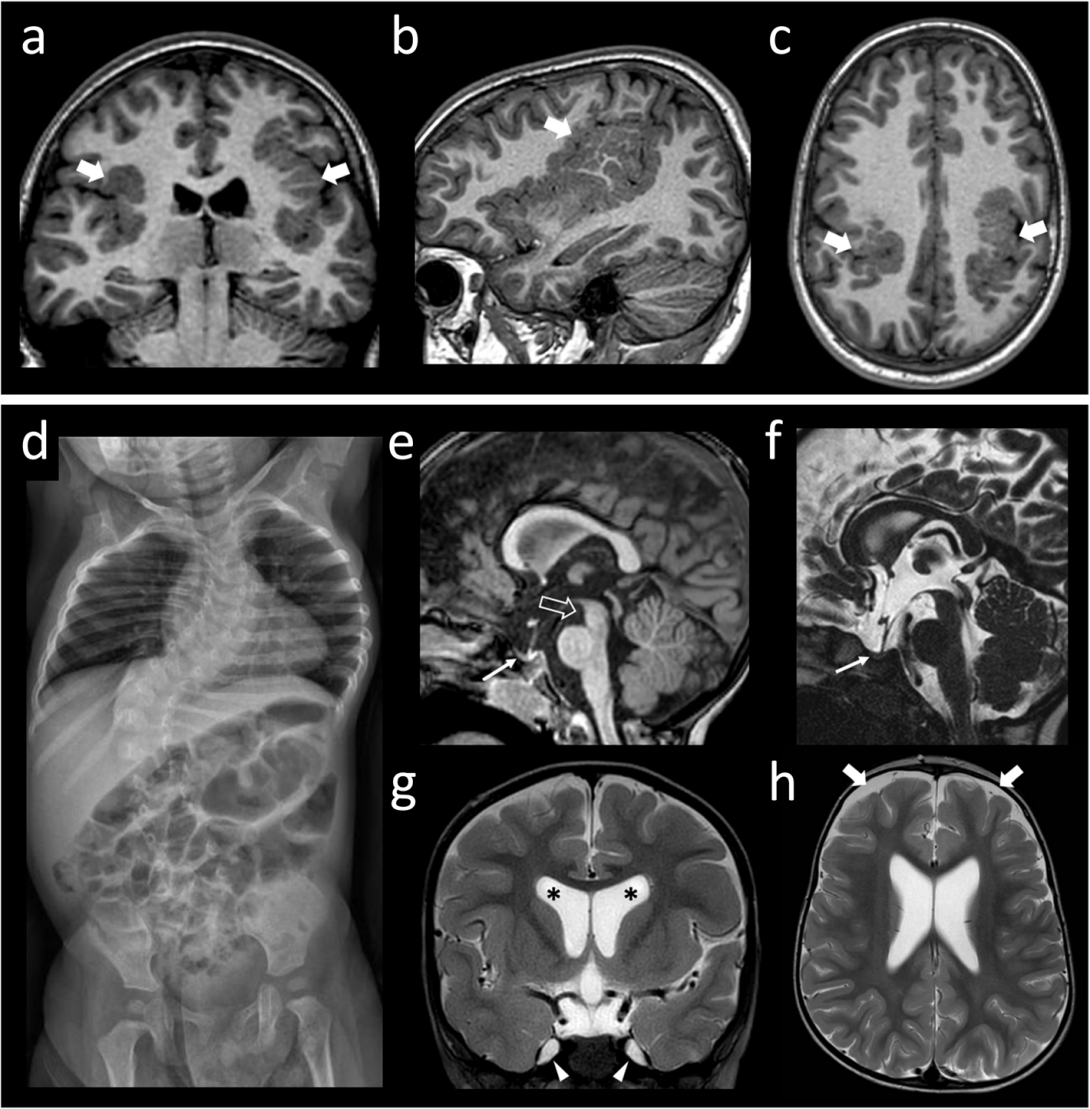
Radiological features. **a**-**c**) Brain MRI of Patient 1 performed at 13 years of age. **a**) Coronal, **b**) sagittal, and **c**) axial 3D T1-weighted reformatted images reveal bilateral polymicrogyria involving the insula and perisylvian regions, slightly more extended on the left side (arrows). **d**-**h**) X-ray and brain MRI of Patient 2 performed at 2 years of age. D) Long-spine plain film in the AP projection shows the severe dorsolumbar scoliosis. **e**) Sagittal T1-weighted and **f**) DRIVE (DRIVen Equilibrium) T2-weighted images demonstrate the hypoplasia of the adenohypophysis (arrows) associated with a minor dysmorphism of the brainstem characterized by a shorter midbrain (empty arrow). **g**) Coronal and **h**) axial T2-weighted images reveal thinning of the periventricular white matter with enlargement of the lateral ventricles (asterisks) and frontal subarachnoid spaces (thick arrows). Note the enlargement of the Meckel's caves (arrowheads)

This boy was referred at age 7 years for epileptic seizures. He was born at term after a regular pregnancy. His parents were non-consanguineous and there was no family history of epilepsy. Neonatal course was uneventful. His developmental history and scholastic performance were normal. General physical and neurologic examination was unremarkable. At the age of 7 years, the child experienced two episodes of tonic-clonic seizures during sleep. Conventional EEGs revealed inter-ictal repetitive spikes in the left temporal area. He started carbamazepine (200 mg two times a day) with very good seizures control, as he became seizure-free. Five years later carbamazepine was slowly discontinued. A few days after the last dose, he experienced a tonic seizure while he was sitting at the table soon after dinner. Carbamazepine 200 mg two times per day was thus restored and no recurrence of seizures was observed in the following 6 months. Afterwards, seizures restarted. A few minutes after starting the meal, he experienced a strong uneasiness followed by behavior arrest, jaw hypotonia and loss of awareness with spontaneous recovery in 1–2 min. He did occasionally experience tonic-clonic seizures no related to food intake or meals. His EEGs showed inter-ictal bilateral mid-temporal epileptiform discharges with increased activity during sleep. Video-EEG monitoring during lunch induced a brief seizure characterized by fixed gaze followed by jaw hypotonia associated with diffuse low-amplitude fast activity followed by generalized spikes and sharp waves mainly evident on mid-temporal areas bilaterally (Fig. 1a, b, c). Brain MRI revealed bilateral perisylvian polymicrogyria (Fig. 2a-c). Array comparative genomic hybridization (aCGH) was unremarkable. The patient was treated with several anticonvulsant agents including carbamazepine (up to 1000 mg daily), valproic acid (up to 1300 mg daily), clonazepam (1 mg three times a day), clobazam (10 mg three times a day), perampanel (up to 8 mg daily), topiramate (up to 250 mg daily), levetiracetam (up to 3000 mg daily) and lacosamide (up to 400 mg daily) with partial benefit. Clonazepam (1 mg three times a day) 30 min before meals together with lacosamide (200 mg twice/day), topiramate (250 mg daily), and valproic acid (500 mg twice/day) resulted quite effective to control his epilepsy, except for a few brief absence seizures with eyelid myoclonus during the day (not meal-related).

### Patient 2

A 2-year-old boy was evaluated for dysmorphic features, history of early-onset recurrent seizures, and brain malformations. He was the first-born from a dichorionic diamniotic twin pregnancy, conceived by intracytoplasmic sperm injection (ICSI) and complicated by intrauterine growth retardation (IUGR) in the third trimester. The baby was delivered at 37 + 1 weeks of gestation by caesarean section. His parents were nonconsanguineous. The twin sister was healthy. Family history revealed a maternal second-degree cousin with epilepsy but was otherwise unremarkable. Birth weight was 1710 g (− 2.8 SD), length 43 cm (− 2.4 SD) and occipitofrontal circumference (OFC) 31.5 cm (− 1.6 SD). Physical examination showed diffuse hypotonia, partially improving during the perinatal period. Dysmorphic features included frontal bossing, highly arched eyebrows, bilateral ptosis combined with blepharophimosis, epicanthus, down-slanting palpebral fissures, micrognathia with tent-shaped mouth and thin upper lip, and low-set posteriorly rotated ears. Brain ultrasonography was normal. At 6 months, global developmental delay was noticed, with persistent head lag and hypotonia with severe thoracolumbar scoliosis (Fig. 2d). From age 7 months, the boy experienced two isolated tonic-clonic seizures. Interictal EEG revealed high voltage spikes and waves complexes over the mid-temporal regions, with increased activity during sleep. Brain MRI showed lack of periventricular white matter bulk with mild enlargement of lateral ventricles and subarachnoid spaces. Furthermore, anterior pituitary hypoplasia and dysmorphic brainstem with midbrain hypoplasia were identified (Fig. 2e-h). aCGH revealed a heterozygous 4.8 Mb deletion extending from 15q25.3 to 15q26.1. This rearrangement spanned 83 genes, including 39 OMIM genes (*ABHD2, ACAN, AEN, ANPEP, AP3S2, BLM, C15orf38, CHD2, CIB1, CRTC3, DET1, FANCI, FES, FURIN, IDH2, IQGAP1, ISG20, KIF7, MAN2A2, MESP1, MESP2, MFGE8, MRPL46, MRPS11, NGRN, PEX11A, PLIN1, POLG, PRC1, RGMA, RHCG, RLBP1, SEMA4B, SLCO3A1, ST8SIA2, SV2B, TICRR, UNC45A, VPS33B*). At age 24 months, during or immediately after eating, the patient suffered from recurrent seizures characterized by staring and upward eye deviation and eyelid myoclonus. The seizures were very short (2–3 s) but occurred in cluster lasting 2–3 min. Ictal EEG revealed a brief discharge of high-voltage spikes and sharp waves mainly evident on mid-temporal areas bilaterally (Fig. 1d). Therapy with sodium valproate (300 mg daily) and topiramate (75 mg daily) did not allow complete seizures control. Furthermore, topiramate was discontinued due to hyporexia and levetiracetam (700 mg daily) was started, with partial seizures control.

## Discussion and conclusions

Reflex epilepsies constitute 2–6% of all epilepsy cases. They are classified as a syndrome in which epileptic events are triggered by specific motor, sensory, or cognitive stimulation. Reflex seizures usually occur in association with spontaneous seizures. As highlighted by Irmen et al. [7], the stimulus can be considered a reflex trigger only if it is strongly associated with the seizure. However, the probability that a certain trigger may elicit a seizure is lower than 100%. Although a specific stimulus can be assumed to act via known epileptogenic mechanisms or hyperexcitable networks, its triggering activity may depend on its degree of interference with the epileptogenic area. EE includes patients “who had more than 50% of fits during or within 30 min of eating breakfast, lunch or dinner” [8]. Eating is a complex phenomenon, mainly involving psychic, sensory, proprioceptive, enteroceptive, and somatosensory stimuli [2, 3]; hence, the exact pathophysiological mechanism by which eating seizures could be induced remains multifaceted. Case reports seem to stress that the action of eating may not solely act as a trigger and also chewing, swallowing, but the context of eating, the mere sight of food or the content of the meal may play a role (see Table 1). Reflex seizures provoked by eating are rare except for specific countries, e.g. Sri Lanka. Seneviratne et al. [1] found one or more provoking factors causing reflex seizures in 47 out of 526 patients (8.9%) from the epilepsy clinic in Ratnapura General Hospital. Eating was the commonest provoking factor, being observed in 28 patients (59.6%). In a prospective study on 1287 epileptic patients seen at Peradeniya, 223 (17.3%) were found to have reflex epilepsy and eating was the commonest stimulus (191 patients, 85.7%) [17]. The very high prevalence of EE in Sri Lanka [1, 17], could be related to genetic or ethnic factors, as well as to the bulky meals rich in carbohydrates consumed by these patients. The high prevalence of EE in Asia is probably due to rice-made foods and heavy meals in such cases, a different mechanism is likely to be implicated such as gastric distention through a vagal reflex. However, familial clustering of a shared genetic background could not be excluded [5]. Out of Sri Lanka, EE is very rare and more prevalent among young adults, even if neonatal cases have been described [9]. EE usually starts in the second-third decade of life, with a male predominance (Table 1). A family history of epilepsy may be present, including siblings suffering from eating seizures as well [5]. Eating can be reported as the only provoking factor or mixed with others, as eating-induced seizures are usually associated with symptomatic epilepsies [4]. Seizures can occur in the early phase of eating (generally within the first 5 min) or in the middle or end of meal [6]. Focal seizures with impaired responsiveness are the commonest seizure type followed by focal seizures without impaired responsiveness and with secondary generalization [6]. Seizures usually arise in temporal-limbic or extra-temporal perisylvian regions and may progress to secondary generalization. EEG often shows focal spikes and sharp/slow waves in the temporal areas. Some patients show a lesional aetiology at neuroimaging; nearly 30 individuals with a likely genetic cause have been reported (Table 1). However, despite the aetiology, the large majority of the patients show EEG patterns consistent with the diagnosis of temporal lobe epilepsy [3, 12, 13, 15]. The physiopathological mechanisms of EE are complex. The temporal lobe is interconnected with the amygdala function and the amygdala with its lower seizure threshold and propensity to induce masticatory seizures may probably be a likely target for stimulation by the oral and masticatory factors [16]. When cases report the mere sight of food or sensory stimuli being able to elicit seizures, the temporal lobe, through the olfactory and gustatory stimuli, may again be involved [14]. Two critical functional cortical loops are likely to be involved: the interconnections between the gustatory cortex (insular, parietal and frontal region) and the frontal-insular-hippocampal network [18] or the frontal-opercular area related to oral movements [19, 20]. In patients with cortical malformations involving these areas, repeated stimuli (such as eating) could induce the hyper-activation of these areas due to the hyperexcitability secondary to the cortical lesion. EE was also described in some rare genetic syndromes [2, 10, 11, 21]. In some genetic epilepsies (e.g., *SYNGAP1* encephalopathy, *MECP2* duplication syndrome), the electroclinical profile correlates with the dysfunction of a limited cortical region (especially the frontal-central cortex), despite the genetic alteration affects the entire brain. The emotional or autonomic components of eating, along with gastric distension or stimulation of the mouth or pharynx, may also play an important role in inducing seizures [22–24]. Monotherapy or polytherapy were used; the most effective treatments reported were sodium valproate, carbamazepine, clobazam and phenobarbital [6]. Monotherapy with carbamazepine likely offers the best seizure control in focal seizures [1]. Patients with “generalized” reflex seizures usually benefit from epileptic drugs typically used for idiopathic generalized epilepsy, such as valproic acid, lamotrigine, or levetiracetam [22]. In some cases, clobazam before meals showed to be effective in preventing eating-related seizures [3, 5]. Some patients need combination therapy. Surgical treatment may be considered when seizures are intractable. In the patient reported by Blauwblomme et al. [18] selective resection of a small region of the insula resulted effective in seizure control. Gujjar et al. [25] further reported a patient with refractory EE who was successfully treated with a temporal lobectomy. The prognosis of reflex seizures is variable due to wide clinical heterogeneity and mostly depends on the nature of the underlying seizure disorder [22]. Both our patients showed additional unprovoked seizures, which is consistent with the findings that unprovoked seizures do occur in EE. In our patients, refractory epilepsy with poor response to multiple antiseizure medications was observed*.* Neuroimaging in patient 1 revealed a cortical bilateral dysplasia involving mid-temporal areas which contain bilateral representations of the face, mouth, and throat. They are also responsive to taste, touch, and proprioception from the tongue and mouth. Excitation of these areas is a normal phenomenon caused by eating, but the presence of structural lesions likely leads to a hyperexcitation fostering the epileptogenesis [26]. The dysfunction of specific cortical regions in the context of a germline genetic alteration might account for the finding of reflex epilepsy in some genetic syndromes, as shown by patient 2. In this case, a relevant contribution to the neurological phenotype is provided by the haploinsufficiency of disease genes involved in the 15q25.3-q26.1 deletion. In particular, de novo loss of function variants in *CHD2* cause an early-onset epileptic encephalopathy and *CHD2* haploinsufficiency in our patient might therefore explain the neurodevelopmental disabilities, dysmorphic features, hypotonia, and complex epileptic phenotype with early onset, mixed, and drug-resistant seizure [27]. Similarly, de novo loss of function variants in *POLG* cause autosomal dominant progressive external ophthalmoplegia (PEO) type 1, a severe neurological condition characterized by progressive weakness of extraocular muscles, proximal or generalized myopathy, and neurological features (e.g., ataxia and parkinsonism) [28]. Accordingly, *POLG* haploinsufficiency might contribute to the neurological and neuromuscular phenotype observed in patient 2. Further research will help clarify the aetiopathogenesis of reflex seizures and shed light on the mechanisms underlying EE, to improve the diagnosis and the management of these rare conditions.

**Table 1:** Review of clinical, imaging, and genetic aspects of EE patients

authors, year [Ref]	n° of EE cases	mean age at eating sz onset (y)	sex (M/F)	trigger stimuli	imaging findings	genetic analysis
Singh et al., 2019 [29]	12	13.5 y	11 M; 1 F	eating alone (75%); eating + anxiety; eating + bathing; eating + spontaneously	normal (7 pts); + 5 pts. (41.6%), focal/bilateral sclerosis or gliosis	na
von Stülpnagel et al., 2019 [2]	8	6.9 y	4 M; 4 F	biting; eating; chewing; oral sensory stimuli	normal	*SYNGAP1* mutations
Jagtap et al., 2016 [4]	47	14.3 ± 9.8 y	41 M; 6 F	eating; eating rice made food; oral sensory stimuli	+ 16 pts. (34%), mainly PC lesions	na
Yacubian et al., 2014 [30]	3	15 y	3 F	eating (independently of type of food)	normal	probably genetic due to familial clustering, but tested negative
Sillanpää et al., 2014 [9]	1	0 y	F	breast feeding	normal	na
Patel et al., 2013 [31]	6	11.3 ± 2.16 y	3 M; 3 F	eating; eating rice made food; “thinking of eating”	+ 5 pts. (83.3%), perysilvian F lobe and high F lesions	na
Kokes et al., 2013 [32]	6	20.3 y	4 M; 2 F	chewing; swallowing; oral sensory stimuli	+ 4 pts. (66.7%), L hemisphere lesions	na
de Palma et al., 2012 [10]	1	6 y	M	eating; oral and gustatory sensory stimuli (mainly spicy food)	normal	*MECP2* duplication
Roche Martínez et al., 2011 [11]	1	16 y	F	eating (independently of type of food)	normal	Rett syndrome but *MECP2, CDKL5, FOXG1* tested negative
Bae et al., 2011 [12]	2	39.5 y	1 M; 1 F	eating (independently of type of food)	normal	na
d’Orsi et al., 2007 [33]	1	25 y	M	chewing; eating; swallowing	+ bilateral opercular dysplasia	na
Loreto et al., 2000 [13]	3	22.7 y	2 M; 1 F	eating; sensory stimuli	+ 2 pts. (66.7%), not specific	na
Mandal et al., 1992 [34]^a^	20	na	16 M; 4 F	eating; eating Indian, rice made food/ heavy meals	normal (in 7 pts. tested)	na
Koul et al., 1991 [35]^a^	78	na	na	eating; swallowing	na	na
Senanayake, 1990 [5]	20	20 y	na	eating	na	probably genetic due to familial clustering
Koul et al., 1989 [14]	50	23.8 y	na	chewing; eating rice made food; swallowing	na	na
Loiseau et al., 1986 [36]	2	20.5 y	2 M	chewing (mainly); eating	na	na
Nagaraja et al., 1984 [15]	13	14 y	8 M; 5 F	chewing; eating Indian, rice made food/heavy meals; drinking	na	na
Aguglia et al., 1983 [3]	3	21.3 y	2 M; 1 F	chewing; eating (independently of type of food)	na	na
Ahuja et al., 1980 [16]	3	21.7 y	3 M	eating (only at home in 2 cases; both at home and outside in 1 case)	na	na
Robertson et al., 1979 [37]	1	14 y	M	eating (independently of type of food)	+ internal capsule astrocytoma (involvement of the right caudate nucleus)	na
Cirignotta et al., 1977 [38]	1	16 y	F	eating (independently of type of food)	na	na
Scollo-Lavizzari et al., 1967 [39]	1	12 y	M	chewing; eating; swallowing; sensory stimuli	na	na

*EE* eating epilepsy, *F* female, *F* frontal, *L* left, *M* male, *n* number, *na* not available, *PC* posterior cortex, *pts*. patients, *sz* seizures, *y* years

^a^only Abstract available

## Data Availability

The data or materials are available from the corresponding author on reasonable request.
